# Public health preparedness in Alberta: a systems-level study

**DOI:** 10.1186/1471-2458-6-313

**Published:** 2006-12-28

**Authors:** Douglas Moore, Alan Shiell, Tom Noseworthy, Margaret Russell, Gerald Predy

**Affiliations:** 1 Centre for Health & Policy Studies, Dept. of Community Health Sciences, University of Calgary, 3330 Hospital Dr. NW, Calgary, AB T2N 4N1, Canada; 2 Dept. of Community Health Sciences, University of Calgary, 3330 Hospital Dr. NW, Calgary, AB T2N 4N1, Canada; 3 Capital Health Region, University of Alberta, Edmonton, AB, Canada; 4 3875 St. Urbain, Bureau 3-02, Montreal, QC H2W 1V1, Canada

## Abstract

**Background:**

Recent international and national events have brought critical attention to the Canadian public health system and how prepared the system is to respond to various types of contemporary public health threats. This article describes the study design and methods being used to conduct a systems-level analysis of public health preparedness in the province of Alberta, Canada. The project is being funded under the Health Research Fund, Alberta Heritage Foundation for Medical Research.

**Methods/Design:**

We use an embedded, multiple-case study design, integrating qualitative and quantitative methods to measure empirically the degree of inter-organizational coordination existing among public health agencies in Alberta, Canada. We situate our measures of inter-organizational network ties within a systems-level framework to assess the relative influence of inter-organizational ties, individual organizational attributes, and institutional environmental features on public health preparedness. The relative contribution of each component is examined for two potential public health threats: pandemic influenza and West Nile virus.

**Discussion:**

The organizational dimensions of public health preparedness depend on a complex mix of individual organizational characteristics, inter-agency relationships, and institutional environmental factors. Our study is designed to discriminate among these different system components and assess the independent influence of each on the other, as well as the overall level of public health preparedness in Alberta. While all agree that competent organizations and functioning networks are important components of public health preparedness, this study is one of the first to use formal network analysis to study the role of inter-agency networks in the development of prepared public health systems.

## Background

International and national events have brought critical attention to the Canadian public health system and how prepared the system is to respond to various types of contemporary public health threats. Whether those threats result from emerging infectious diseases or bioterrorism, the public health system is responsible for protecting the health of the population. Outbreak response occurs first at the local or regional levels and thus the potential impact that a threat will have on the overall Canadian population can differ significantly depending on the capacity of public health systems to respond[1]. For example, following the SARS outbreak in Toronto, the Canadian National Advisory Committee (NAC) on SARS and Public Health held it as fortunate that the SARS outbreak struck primarily in Toronto and not in other parts of Canada where the capacity to combat public health threats is limited[2]. While a "general renewal of the public health infrastructure" and its capacity to respond are in order the effective coordination of all agencies at regional, provincial, and federal levels is necessary to assure a comprehensive surveillance and management of public health threats. As Ralph Klein, the Premier of Alberta, commented: "Albertans and all Canadians understand a disease outbreak like SARS or West Nile virus in one region affects other parts of the country. Coordinated approaches will help deal with public health threats."

Despite the commitment of federal and provincial agencies in Canada to improve public health system performance and develop overall public health preparedness, little is still known about the current systems-level state of public health preparedness in Canada. While coordination is seen as a necessary element for public health preparedness, few measures of inter-organizational collaboration relevant to public health preparedness have been developed and assessed in relation to other features of the organizational and inter-organizational environment. This study protocol presents the premises, conceptual model and methods used to measure and assess public health preparedness in Alberta, Canada. Although provincial public health agencies are themselves embedded within federal and global public health preparedness systems, we focus primarily on the organizational environment of public health preparedness in Alberta so as to develop contextually-relevant measures and evaluation techniques as well as address issues related to potential cross-Alberta variations in public health preparedness.

In Alberta, public health and emergency response procedures are primarily governed under the *Disaster Services Act *[3] and the *Public Health Act*[4]. Provincial response to a public health disaster event may thus call into action organizations and units under the jurisdiction of local municipalities, regional health authorities, emergency management districts, and/or the provincial government. There are a number of organizational actors that may be involved in preparation, response, or recovery phases of a public health disaster, including departments or portfolios within agencies. Each of which are potential actors in the provincial public health and emergency response network. While we consider the overall public health and emergency management network to consist of all potential organizational actors involved in responding to public health threats, the precise set of organizations that come together around a particular public health threat depends on the nature of that threat. For example, pandemic influenza and the West Nile virus are both emerging infectious diseases but the nature of their transmission differs and distinctive sets of organizational actors must be in place to confront them. In the case of West Nile Virus, Alberta's Department of the Environment and Sustainable Resource Development would be integrally involved for mosquito surveillance and control whereas it would not be as integral in the event of a pandemic influenza outbreak[5]. To assess potential differences by public health threat in Alberta public health preparedness, we examine and compare two potential public health threats: pandemic influenza and West Nile Virus.

## Methods/Design

### Conceptual model

**Figure 1:**
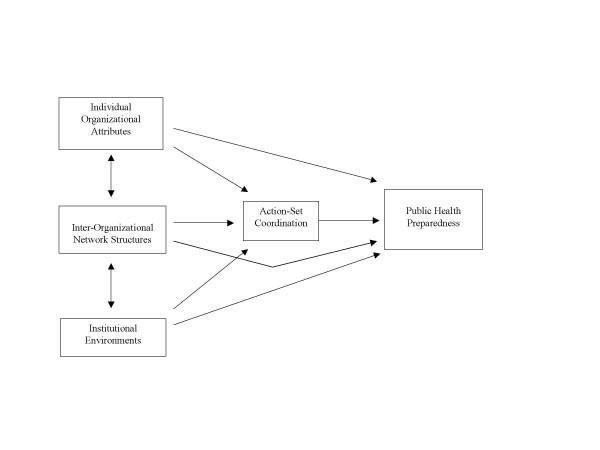
PHP Systems Conceptual Framework.

Using an embedded multiple-case study design[6] and integrated qualitative and quantitative methods, the project proposes to measure empirically the degree of seamless coordination existing among municipal, regional or district-level and provincial public health and emergency management agencies in Alberta. Although federal authorities and agencies play an important role in provincial-level responses to public health emergencies, for the purposes of this research, the focus will be on the inter-organizational linkages and organizational environments of provincial and sub-provincial actors; relevant federal-level organizations will be identified for future investigation. The seamless coordination of activities and tasks that should characterize the overall federal-provincial-regional linkages should also characterize the intra-provincial linkages. Fluid communication and resource flows among such diverse organizations and administrative authorities require an integrated and coordinated network of actors. Our research is premised on empirical research that has shown that effective and coordinated approaches to public health threats require a well-integrated and responsive inter-organizational system and that the most appropriate method in which to analyze inter-organizational relations and the degree of integration is one based on network analysis [7]. Yet, strong inter-organizational ties are not enough for effective preparedness if the organizations involved do not have the necessary individual capacity to respond to public health threats. In this regard, fluid communication and integrated organizational connections are but one element in an overall systems-level approach to assessing public health preparedness. As shown in Figure 1, we view public health preparedness to be a product of several interrelated factors: 1) individual organizational attributes, 2) the inter-organizational networks existing among relevant organizational actors, 3) the institutional environments in which organizations and inter-organizational networks are located and in which threats first emerge, and 4) the integrated coordination of specific action-sets. An action-set refers, in this case, to a subgroup of organizations in the network that come together purposefully to address a specific public health threat. We see these four factors together influencing system-level public health preparedness.

### Sampling design

Our target population for the research is all public health and emergency management-related organizations in Alberta. The construction of the project's sampling frame is based on a stratified, multistage cluster design. The strata are four administrative divisions: (1) provincial-level agencies, (2) sub-provincial administrative agencies, i.e., emergency-management districts (disaster services) and regional health authorities, (3) metropolitan areas, and (4) towns and local areas. Within strata 1–3, the sampling frame includes all public health or emergency management organizations, i.e., agencies, departments, or units, within those strata. The fourth stratum will be sub-stratified according to the Alberta Emergency Management district divisions into 6 geographical areas: (1) northwestern Alberta, (2) northeastern Alberta, (3) north-central Alberta, (4) central Alberta, (5) south-central Alberta, and (6) southern Alberta. From each of the 6 geographical regions, we will draw a proportionate, random sub-sample of towns. For stratum 4, the sampling frame consists of all public health or emergency management-related organizations in those towns randomly selected. The project's frame population will thus consist of those organizations listed in the strata 1–3 sampling frame and those listed in the stratum 4 sampling frame.

### Phase one: initial qualitative research

This study will be conducted in 2 phases: phase 1 qualitative and phase 2 quantitative research. Phase 1 is used in part to help identify the organizations responsible for public health and emergency responses and the relevant relationships among them. Initial qualitative research will consist of the analysis of official documents and reports and interviews with key organizational representatives. Government documents and reports will be analyzed to 1) help identify agencies involved in the response system, 2) determine the formal tasks assigned to those agencies, and 3) initially assess the overall level of preparedness and responsiveness for the three case studies. Interviews with key informants and health officials will be used to determine 1) if there are additional organizations involved in the response system that were not noted in the official documents, 2) the types of relationships characterizing the inter-organizational ties within and among action-sets, and develop 3) relevant measures of the institutional environment and of public health preparedness and responsiveness. While laws mandate that certain organizations be involved in emergency responses to specific public health threats, key organizational representatives may identify agencies that play an important albeit informal role in local emergency response systems.

In addition to helping identify organizations that may be absent from official documents, the interviews will also investigate the perceived characteristics of inter-organizational relationships. Mandated relationships, for example, tend to be characterized by lower levels of perceived cooperation among organizations [8]. While such characteristics will be investigated using quantitative methods in phase 2, the qualitative research will reveal the points of conflict and cooperation that emerge in the course of public health and emergency responses and thus provide a basis for the questions that will be used in the organizational questionnaire. The final focus of the phase one interviews will centre on the development of measures of the institutional environment, network effectiveness, and public health preparedness. What are the resources that key agency representatives themselves identify as critical and important to their organization and the network? What processes do the respondents use to evaluate if their organization or the system was prepared?

In phase 1, we will draw a non-proportional sample from the project's sampling frame. We will interview 6 organizational representatives from the provincial level (stratum 1), 12 representatives from the sub-provincial level (strata 2 and 3), and 18 representatives from the local level (stratum 4). Phase 1 interview respondents will be organizational representatives who are randomly selected from each of these strata and choose to participate.

Interviews will be tape-recorded and transcribed so that content analysis, thematic, and inter-textual analyses can be conducted on the interviews. Transcripts will be coded for such indicators as (1) organizations involved in the public health and emergency management preparedness, (2) types of information-sharing among organizations, (3) types of resource flows existing among organizations, and (4) perceptions of local and provincial public health preparedness. Content analysis will be used to identify relevant organizations that were not identified in the documentary research [9,10]. Thematic analyses will be used to identify the critical themes emerging in the 36 interviews; inter-textual analyses will be used to compare the responses of interviewees across the three strata dimensions of jurisdiction, domain, and geographical location.

### Phase two: organizational questionnaires

Phase 2 involves the administration of an organizational/inter-organizational questionnaire to Alberta-based public health and emergency management agencies. Information on organizations and inter-organizational networks will be collected by web-based or telephone questionnaires. For phase 2, we intend to administer questionnaires to representatives of all organizations listed in the project's sampling frame (described earlier). The questionnaire consists of three components: 1) an organizational attribute component, 2) an inter-organizational network component, and 3) an organizational environment and network assessment component. The organizational attribute component consists of questions on the general characteristics of organizations such as staff size, training background of specific occupational roles in the organization, sectoral operations, budget and more emergency-response related questions, such as whether an agency has formally assessed its epidemiology capacity.

The inter-organizational network component consists of items that ask representatives to identify those organizations with which their own organization has ties along specific dimensions. The specific content and dimensions of the network to be mapped will be determined following phase one analysis but candidate dimensions used in other organizational network studies include (i) information-sharing, (ii) resource-sharing, including staff and (iii) joint-planning. For each of the critical dimensions identified in phase one, the respondent will be presented with a list of organizations within the relevant action-set and asked, for each one, "who do you *share information *with?" or "who do you *share resources *with."

The third component of the questionnaire will consist of the respondent's subjective assessments of the institutional environment and inter-organizational network, and the impact that each has on perceived public health preparedness. For example, how do representatives assess the environment in which they operate, i.e., do they see resources as easily available or scarce? How prepared do they perceive their own organization and local system to be for a public health threat? Since mandated relations involve "sequential interdependence," an important question is whether organizations perceive the task transitions among organizations as functioning smoothly. The actual questions that will be used in this component of the questionnaire will be determined following phase one data analysis.

Ethical approval of the research has been granted by the Conjoint Health Research Ethics Board of the Calgary Health Region and University of Calgary, Faculty of Medicine (ID#17873) and of the Capital Health Region and University of Alberta (#B-251005).

## Discussion

The capacity of any organization to be prepared for a public health threat is influenced in part by the system in which the organization is located, just as the preparedness of the overall system can be influenced by the strengths and weaknesses of any single organization. The cross-case and intra-provincial comparative design allows our research to discriminate among different system elements and examine the independent influence of organizational attributes, institutional environments, and inter-organizational networks on public health preparedness. In addition, the mixed methods approach enables our research to analyse the role of both informal and formal relationships in the emergence of inter-organization networks and the development of prepared and responsive public health systems. As far as we are aware, our project is one of the first to assess public health preparedness at the systems-level while accounting for the critical role that inter-organizational relationships play in the emergence of a cohesive and responsive system. Moreover, in deciphering the independent influence of inter-organizational ties within the overall system, the research will provide measures that will help researchers and policy-makers evaluate the degree of coordination among organizational actors. Where are the gaps in system linkages? Which organizations need to be made more central to preparedness activities? These are just a few of the questions that our research is designed to answer.

## Competing interests

The author(s) declare that they have no competing interests.

## Authors' contributions

All authors participated in the design of the study. DM coordinated the study and drafted the manuscript. All authors read drafts of the manuscript, made comments and suggestions, and approved the final version.

## Pre-publication history

The pre-publication history for this paper can be accessed here:
